# The Double-Edged Nature of Methyl Donors in Cancer Development from Prevention to Progression

**DOI:** 10.3390/ijms27010323

**Published:** 2025-12-28

**Authors:** Da Pan, Shaokang Wang, Guiju Sun

**Affiliations:** Key Laboratory of Environmental Medicine and Engineering, Ministry of Education, Department of Nutrition and Food Hygiene, School of Public Health, Southeast University, Nanjing 210009, China; shaokangwang@seu.edu.cn

**Keywords:** one-carbon metabolism, methyl-donor nutrients, cancer prevention, dual-role hypothesis, epigenetic regulation, cancer progression, precision nutrition

## Abstract

Methyl-donor nutrients, including folate, vitamin B12, vitamin B6, choline, betaine, and methionine, play indispensable roles in one-carbon metabolism and govern key processes such as DNA methylation, nucleotide synthesis, and genomic maintenance. Yet despite decades of research, their relationship with cancer remains paradoxical and frequently misunderstood. Much of the confusion arises from an overreliance on epidemiological studies that use cancer incidence as a late-stage endpoint, thereby obscuring how the biological actions of methyl donors differ fundamentally across the continuum from precancerous lesions to established tumors. By synthesizing evidence from mechanistic studies, precancerous lesion research, and early-stage carcinogenic models, this review suggests that adequate methyl-donor availability may be protective during the earliest phases of cancer development. However, these same nutrients may later become substrates hijacked by neoplastic cells to fuel rapid proliferation, maintain oncogenic methylation programs, and enhance tumor progression in established malignancies and high-risk populations. Therefore, this review proposes a reframing that methyl donors may not be evaluated merely as protective or harmful, but rather as context-dependent modifiers whose influence is shaped by timing, metabolic status, and the underlying biology of the target tissue. Such a shift is promising for advancing precision nutrition and the prevention or targeted suppression of cancer.

## 1. Introduction

Methyl donors—such as folate, vitamin B12, vitamin B6, choline, betaine, and methionine—are essential nutrients that fuel one-carbon metabolism and drive methylation reactions across the genome [1]. These reactions play a central role in maintaining epigenetic homeostasis, genomic stability, and cellular integrity [2,3]. Through the transfer of methyl groups, methyl donors regulate DNA methylation, RNA processing, and histone modification, thereby influencing gene expression patterns that determine cell fate, differentiation, and response to environmental stimuli [4].

Aberrant methylation has long been recognized as a hallmark of cancer. Global hypomethylation can activate oncogenes and destabilize chromosomal architecture, whereas regional hypermethylation may silence tumor suppressor genes [5]. Given their role in modulating methylation capacity, nutritional methyl donors have emerged as critical dietary factors that can either prevent or promote tumorigenesis, depending on context and timing [6]. Accumulating evidence suggests a potential biphasic role of methyl donors during the cancer continuum. For instance, researchers proposed a hypothesis based on conflicting research findings [7], suggesting that in the early stages, adequate methyl donor availability supports DNA repair, limits mutation accumulation, and maintains normal epigenetic programming—thereby exerting protective effects against malignant transformation. Conversely, once neoplastic cells are established, excessive methyl donor supply may enhance methylation-dependent silencing of key regulatory genes, promoting tumor cell proliferation, invasion, and metastasis. Thus, the same nutrients that guard genomic stability in healthy tissues may, under certain conditions, fuel cancer progression [6].

This dualistic behavior highlights the complexity of nutritional–epigenetic interactions in cancer biology. Although this hypothesis has not yet been definitively verified and no solid conclusion has been reached within the scientific community, understanding how methyl donors influence distinct phases of carcinogenesis—initiation, promotion, and progression—will be essential for optimizing dietary recommendations, designing targeted interventions, and identifying windows of therapeutic opportunity. This review aims to summarize current evidence on the mechanistic and clinical implications of methyl donor metabolism in cancer, emphasizing its context-dependent, double-edged nature.

## 2. Overview of Methyl Donors and One-Carbon Metabolism

### 2.1. Key Nutrients and Pathways

Methyl donors are a group of interrelated nutrients that sustain the biochemical framework of one-carbon metabolism—a network of reactions essential for DNA synthesis, amino acid interconversion, redox homeostasis, and epigenetic regulation. The major participants in this network include folate, vitamin B12, vitamin B6, methionine, choline, and betaine, each contributing unique yet complementary roles in transferring and recycling one-carbon units [8].

**Figure 1:**
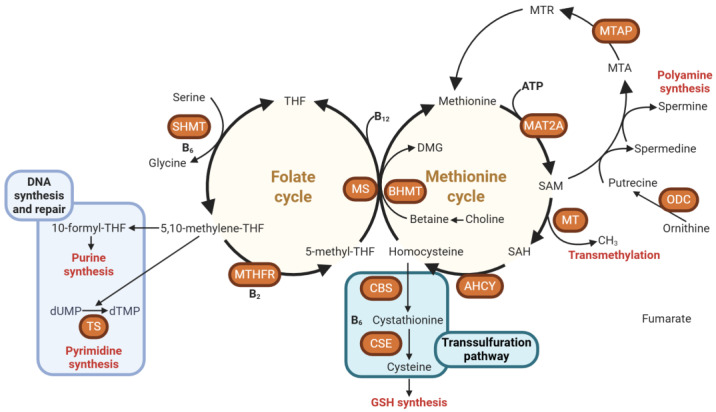
Schematic representation of the one-carbon metabolism. The enzymes are highlighted in Orange. Tetrahydrofolate (THF), serine hydroxymethyltransferase (SHMT), 5,10-methylenetetrahydrofolate (5,10-methylene-THF), methylenetetrahydrofolate reductase (MTHFR), 5-methyltetrahydrofolate (5-methyl-THF), methionine synthase (MS), 10-formyltetrahydrofolate (10-formyl-THF), deoxyuridine monophosphate (dUMP), deoxythymidine monophosphate (dTMP), thymidylate synthase (TS), betaine-homocysteine methyltransferase (BHMT), dimethylglycine (DMG), methionine adenosyltransferase 2A (MAT2A), S-adenosylmethionine (SAM), methyltransferases (MT), S-adenosylhomocysteine (SAH), adenosylhomocysteinase (AHCY), cystathionine β-synthase (CBS), cystathionine γ-lyase (CSE), glutathione (GSH), Methylthioadenosine (MTA), methylthioadenosine phosphorylase (MTAP), 5-deoxy-5-(methylthio)ribose (MTR), ornithine decarboxylase (ODC).

Within this metabolic system, two central cycles—the folate cycle and the methionine cycle—operate in close coordination (Figure 1) [8]. The folate cycle revolves around tetrahydrofolate (THF), which acts as a carrier of single-carbon groups in varying oxidation states. Through the enzymatic action of methylenetetrahydrofolate reductase (MTHFR), 5,10-methylene-THF is reduced to 5-methyltetrahydrofolate (5-MTHF), the biologically active form responsible for donating a methyl group to homocysteine. This remethylation step, catalyzed by the vitamin B12–dependent enzyme methionine synthase (MS), converts homocysteine into methionine and regenerates THF, thus maintaining a cyclic flow of one-carbon units. Genetic polymorphisms in MTHFR can diminish enzymatic efficiency, impair methylation capacity, and increase susceptibility to various diseases through the accumulation of homocysteine and reduced S-adenosylmethionine (SAM) synthesis [9].

Methionine, once formed, serves as the substrate for methionine adenosyltransferase (MAT), which catalyzes its conversion into SAM [10]. SAM represents the universal methyl donor in almost all methylation reactions, transferring methyl groups to DNA, RNA, proteins, phospholipids, and neurotransmitters [10,11,12]. After methyl donation, SAM is converted into S-adenosylhomocysteine (SAH), which is subsequently hydrolyzed by S-adenosylhomocysteine hydrolase into adenosine and homocysteine. The balance between SAM and SAH is a sensitive indicator of cellular methylation potential; a high SAM/SAH ratio promotes methylation, whereas accumulation of SAH inhibits methyltransferase activity [13].

Parallel to the folate-dependent route, choline and betaine constitute an alternative methylation system—particularly active in the liver and kidney—mediated by the enzyme betaine-homocysteine methyltransferase (BHMT) [14,15]. In this pathway, betaine donates a methyl group to homocysteine, producing methionine and dimethylglycine (DMG). This reaction becomes especially important under conditions of folate or vitamin B12 deficiency, providing metabolic redundancy to preserve methionine and SAM synthesis [16]. Thus, the interplay between the folate, methionine, and BHMT-dependent pathways ensures both metabolic flexibility and stability of the methylation pool. Additionally, a portion of homocysteine can be diverted into the transsulfuration pathway through the action of cystathionine β-synthase and cystathionine γ-lyase, both requiring vitamin B6 as a cofactor [17]. This branch converts homocysteine into cystathionine and subsequently cysteine, which serves as a precursor for the antioxidant glutathione (GSH). Through this linkage, one-carbon metabolism not only supports methylation but also maintains redox balance, a critical determinant of cellular survival and genomic stability [18,19].

### 2.2. Regulation of DNA and Histone Methylation

Among all cellular metabolites, SAM plays a uniquely central role as the primary methyl group donor for epigenetic reactions. SAM-dependent methyltransferases catalyze the transfer of methyl groups to both DNA and histone substrates, thereby shaping chromatin architecture and regulating transcriptional activity [20,21,22]. DNA methylation involves the covalent addition of a methyl group to the 5′ position of cytosine residues within CpG dinucleotides, forming 5-methylcytosine (5mC). This reaction is catalyzed by a family of DNA methyltransferases (DNMT1, DNMT3A, DNMT3B, and DNMT3L), which use SAM as a methyl donor. Proper DNA methylation patterns are essential for genomic imprinting, X-chromosome inactivation, and suppression of repetitive elements. Aberrant methylation—either global hypomethylation or promoter-specific hypermethylation—can disrupt gene expression programs, contributing to genome instability, aberrant cell proliferation, and oncogenic transformation [4].

Histone methylation provides another layer of epigenetic control. The N-terminal tails of histone proteins, particularly histones H3 and H4, undergo reversible methylation at specific lysine (K) and arginine (R) residues, mediated by histone methyltransferases (HMTs or KMTs) and demethylases (KDMs) [23]. These modifications alter the electrostatic interaction between histones and DNA, switching chromatin between transcriptionally active (euchromatin) and repressed (heterochromatin) states. The effects of methylation depend on the specific residue and the degree of methylation—for instance, trimethylation at H3K4 is associated with active transcription, whereas H3K9me3 or H3K27me3 marks gene silencing [24,25]. Fluctuations in SAM levels, therefore, directly influence the extent of histone methylation and consequently the transcriptional landscape. An imbalance in one-carbon metabolism—whether from nutrient deficiency or excess—can thus propagate into widespread epigenetic reprogramming, a mechanism increasingly recognized in both developmental regulation and cancer progression [8,26,27].

### 2.3. Interaction with Other Nutrients and Cofactors

Beyond the core methyl donors, several auxiliary nutrients act as cofactors to maintain the efficiency of one-carbon metabolism. Vitamin B2 serves as a coenzyme for MTHFR, facilitating the conversion of 5,10-methylene-THF to 5-MTHF, thereby supporting the remethylation of homocysteine [28]. Vitamin B6 is indispensable for the transsulfuration reactions that convert homocysteine to cystathionine, regulating homocysteine concentrations and preventing its toxic accumulation [29]. Zinc functions as a structural element in both DNA-binding proteins and methyltransferases, ensuring proper catalytic activity and chromatin interaction [30,31]. Magnesium, on the other hand, is required for ATP-dependent reactions within the methionine cycle, including the synthesis of SAM by MAT [32]. Deficiency of these cofactors can compromise methylation capacity, leading to altered SAM/SAH ratios, disrupted gene regulation, and increased oxidative vulnerability. Together, these interacting pathways and cofactors form a tightly controlled metabolic system that links nutrient availability to epigenetic fidelity. Disruption at any node of this network—through diet, genetics, or environmental stressors—can propagate into widespread changes in gene expression and cellular phenotype, setting the stage for disease development, including cancer.

## 3. The Dual Role of Methyl Donors in Cancer: From Prevention to Progression

Epidemiological studies have shown inconsistent associations between methyl donor nutrients, particularly folate and vitamin B12, and cancer outcomes, failing to reach a definitive conclusion [33]. This inconsistency may be attributed to the limitations of previous research, most of which primarily focused on cancer as a clinical endpoint, often overlooking the potential varying roles of these nutrients at different stages of cancer initiation and progression [34,35,36,37,38]. This limitation likely leads to results that reflect only the impact of methyl donors at specific stages of carcinogenesis or their combined effects across different phases. Consequently, future research designs must adopt more refined approaches, whether in population studies or mechanistic investigations using animal or cell models. In this review, we likewise focus on discussing studies with more refined designs. Since the contradictory conclusions from epidemiological studies using cancer as a clinical endpoint have already been widely debated [6], it is now more crucial to draw on existing literature to thoughtfully dissect and analyze the deeper mechanisms, and to identify the logical connections underlying these complexities.

### 3.1. Methyl Donors in Cancer Prevention: Safeguarding Genomic Stability Through Epigenetic Regulation

The link between methyl-donor status and carcinogenesis was observed early in animal models. Decades ago, prolonged intake of diets deficient in multiple methyl-donors (including methionine, choline, folate, and vitamin B12) was shown to induce hepatomas in rats and promote chemical carcinogenesis [39]. Investigations into the underlying mechanism revealed that the cancer-promoting effects were preceded by remarkably rapid changes in the epigenome: DNA hypomethylation was detected in the liver within just one week of initiating a methyl-deficient diet, occurring concurrently with increased DNA synthesis and long before tumor formation [39]. This rapid epigenetic disruption provided a strong causal link, suggesting that the chronic failure to fully methylate newly synthesized DNA serves as an early, pivotal trigger for malignant transformation. This foundational understanding laid the groundwork for detailed investigations into individual components of the methyl cycle, focusing initially on the most well-characterized nutrient, folate. Among these methyl donors, folate has been the first to be observed and suspected to have a “dual role” in cancer development [40]. This variability may depend on factors such as the individual’s folate receptor content, duration of intake, and metabolic status [41]. As a result, numerous studies have been conducted to validate this finding and explore the underlying mechanisms, with the goal of leveraging these insights to develop targeted therapies. One such study investigated the effects of folate supplementation on gliomagenesis in rodent models and provided significant insights into the preventive role of folate in cancer initiation [42]. The study showed that folate supplementation reduced tumor volume in rodent models of glioma induced by various methods, including genetic modification and chemical treatment. Mechanistically, folate’s action was linked to its ability to increase global DNA methylation levels in tumor tissues, specifically in DNA repeat elements and oncogenes such as *PDGF-B* and *survivin*, without affecting tumor suppressor genes (TSGs) like *p53* or *PTEN*. This selective methylation pattern suggests that folate supplementation plays a critical role in maintaining genomic stability during the early stages of tumorigenesis. By promoting the methylation of oncogenes and DNA repeat elements, folate appears to prevent chromosomal instability, a key driver of cancer development. The study also highlighted that folate treatment did not induce pro-neoplastic effects in non-cancerous tissues, such as colorectal tissue, where it did not alter the methylation of TSGs or induce tumor formation. This specificity supports the notion that folate’s effects are focused on the prevention of tumor initiation rather than the promotion of malignancy. Furthermore, the study emphasized that folate’s ability to increase DNA methylation in tumors correlated with reduced tumor volume, reinforcing the idea that folate can modulate epigenetic changes to favor a less aggressive tumor phenotype [42]. The observed increase in methylation of DNA repeat elements, such as *LINE-1*, suggests an anti-tumorigenic effect, as hypomethylation of these elements is associated with chromosomal instability [43,44]. Another study investigating the effects of aging and dietary folate on DNA methylation in mouse colon demonstrated that the aged colon is particularly vulnerable to folate availability, and that folate supplementation could reverse age-related epigenetic aberrations. By promoting genomic DNA methylation and modulating gene-specific promoter methylation such as *p16*, folate appears to help maintain an epigenetic environment that is less conducive to carcinogenesis, thereby potentially playing a preventive role in the initiation of cancer, especially in age-related contexts [45]. Further supporting this early-stage preventive effect, recent human evidence has shifted attention toward precancerous lesions. Studies conducted in a high-risk population for esophageal cancer showed that individuals with esophageal precancerous lesions had significantly lower circulating folate levels and exhibited higher frequencies of aberrant promoter hypermethylation in key tumor suppressor genes, including *p16* and *p53* [46,47]. Importantly, higher folate status was associated with a reduced risk of developing these lesions, suggesting that sufficient folate helps normalize early epigenetic disruptions that would otherwise silence critical tumor suppressor pathways. Through restoring proper promoter methylation and maintaining genomic stability, folate may act to prevent the transition from dysplasia to malignant transformation at the earliest detectable stage [46]. This further supports the hypothesis that folate supplementation may serve as a protective mechanism against the initiation of cancer by stabilizing the epigenome and preventing mutagenic changes that could lead to neoplastic transformation.

Compared with folate, research on other methyl-donor nutrients has been far less detailed. In recent years, a few studies have begun to examine the relationship between vitamin B12 status and precancerous lesions, aiming to determine whether vitamin B12 may exert protective effects during the earliest stages of carcinogenesis [47,48]. However, the available evidence remains limited in both number and methodological diversity, relying largely on case–control designs in human populations. Even so, these studies may provide more informative insight than earlier investigations that focused solely on cancer outcomes, and they offer meaningful preliminary clues regarding the potential early-stage role of vitamin B12 in cancer prevention. For example, a case–control study utilizing precise 3-day duplicate diet samples found that while dietary intake of vitamin B12 was not directly associated with the risk of esophageal precancerous lesions (EPL), higher serum levels of vitamin B12 and transcobalamin II (TC II)—the protein responsible for mediating cellular uptake of bio-active vitamin B12—were significantly associated with reduced risk [48]. This discrepancy between intake and serum levels suggests that bioavailability and transport efficiency may be more critical than dietary consumption alone in preventing early-stage carcinogenesis. Expanding on these findings, subsequent research confirmed that vitamin B12 depletion and reduced TC II levels are risk factors for EPL, a relationship likely mediated by the “methyl folate trap” mechanism [47]. In this molecular pathway, vitamin B12 functions as an essential coenzyme and methyl carrier that bridges the folate and methionine cycles: it accepts a methyl group from 5-methyltetrahydrofolate (5-MTHF) to remethylate homocysteine into methionine, which is subsequently converted into SAM, the universal methyl donor required by DNA methyltransferases to maintain genomic stability [49,50]. Consequently, the concurrent elevations of serum homocysteine and 5-MTHF observed in EPL patients serve as biochemical evidence of a blockade in this critical transfer process. This metabolic disruption prevents the efficient production of SAM, thereby destabilizing the epigenetic landscape and facilitating the aberrant DNA methylation patterns associated with tumor initiation [51,52]. Corroborating these mechanistic insights, experimental evidence from animal models has demonstrated that vitamin B12 deprivation drives global hypomethylation even in the presence of a folate-rich diet [53]. These findings indicate the critical necessity of maintaining a synergistic balance between folate and vitamin B12 to ensure the proper catalysis of one-carbon metabolism and the preservation of physiological DNA methylation integrity [54]. Furthermore, this protective role appears to be modulated by gene-nutrition interactions; specifically, the *TCN2 C776G* polymorphism, which influences vitamin B12 availability, was shown to interact with nutritional status, resulting in a drastically increased risk of EPL in males with low serum vitamin B12 levels [47]. Mechanistically, vitamin B12 depletion in these subjects was linked to aberrant DNA methylation patterns characteristic of cancer initiation, including global hypomethylation and region-specific hypermethylation of the *UGT2B15* and *FGFR2* gene promoters [47].

Similarly, vitamin B6 acts as another indispensable cofactor within the one-carbon metabolism network, yet its independent contribution to cancer prevention has historically been difficult to isolate from other dietary factors. Providing clarity to this landscape, a recent comprehensive meta-analysis gathering the largest collection of data on the subject to date—encompassing nearly 100,000 cancer cases—has offered evidence supporting a potential preventive role for vitamin B6 [55]. The results from observational studies revealed a strong inverse association between vitamin B6 status and overall cancer risk, with the most consistent and statistically significant findings observed in gastrointestinal malignancies, particularly colorectal carcinoma. Crucially, this analysis suggested that bioavailability is a key determinant of efficacy: while data on dietary intake showed significant heterogeneity, blood levels of pyridoxal 5′-phosphate (PLP)—the active form of vitamin B6—demonstrated a strong, homogeneous association with reduced cancer risk, particularly for gastrointestinal tumors. This consistency in PLP data suggests that Vitamin B6 likely exerts a direct biological effect against carcinogenesis rather than merely serving as a surrogate marker for a healthy diet [55]. At the molecular level, the protective role of PLP is largely attributed to its essential function in maintaining genomic stability. PLP serves as a required cofactor for numerous enzymes, including serine hydroxymethyltransferase (SHMT), which facilitates the efficient production of one-carbon units necessary for nucleotide biosynthesis and methylation processes [56]. Specifically, vitamin B6 deficiency compromises SHMT activity, leading to a shortage of one-carbon donors (such as 5,10-methylene-THF) and consequently driving the misincorporation of uracil into the DNA strand in place of thymidine. This uracil misincorporation is a critical molecular mechanism that causes DNA strand breaks and impaired DNA repair, which are hallmarks of genomic instability and drivers of malignant transformation. Furthermore, adequate vitamin B6 status contributes to the lowering of circulating homocysteine levels and supports the activity of detoxification enzymes like Glutathione S-transferases, thereby mitigating oxidative stress, reducing inflammatory responses, and inhibiting excessive cell proliferation, providing multifaceted protection against tumor development [57]. The molecular mechanisms linking vitamin B6 deficiency to carcinogenesis are further supported by a spectrum of in vivo and clinical observations. For instance, experimental models consistently demonstrate that PLP deficiency is oncogenic: studies utilizing Drosophila have shown that PLP depletion directly precipitates the development of malignant tumors [58,59,60]. Similarly, in mammalian systems, rodent models have indicated that inadequate vitamin B6 status exacerbates cancer risk: mice fed a deficient diet exhibited an increase in colon tumorigenesis, while deficiency in rats has been linked to signs of chronic pancreatitis, a condition strongly established as a precursor state that significantly elevates the risk of pancreatic cancer [61]. In the human context, clinical evidence also points to the importance of adequate vitamin B6 status, with a high prevalence of vitamin B6 deficiency being reported among patients diagnosed with primary and secondary myelofibrosis [62]. These collective findings from different model organisms and patient populations suggest the critical need for optimal vitamin B6 levels to protect against the initiation of various malignancies. Although meta-analyses of randomized controlled trials (RCTs) have not yet confirmed this preventive effect, these null findings were graded as low-level evidence due to significant methodological limitations, including the confounding co-administration of other vitamins and the fact that cancer outcomes were often not the primary focus of these trials [55]. Consequently, current evidence points toward circulating PLP as a valuable biomarker for cancer predisposition, reinforcing the necessity of maintaining optimal B-vitamin status to safeguard genomic stability.

As methyl donors, choline and its oxidized metabolite betaine are distinct from the B-vitamin family yet play an equally indispensable role in maintaining epigenetic homeostasis. Mechanistically, betaine provides methyl groups through the BHMT pathway to remethylate homocysteine into methionine, thereby sustaining intracellular SAM pools required for DNA and histone methylation [14,15]. Similar to other methyl donors, epidemiological studies on choline and betaine have produced heterogeneous findings—including inverse, null, and even positive associations with cancer outcomes—a pattern that likely reflects the methodological limitations inherent in studies relying on cancer incidence as a late-stage endpoint [63,64,65]. In contrast, mechanistic evidence offers a far clearer perspective, demonstrating that adequate choline and betaine status protects against early carcinogenesis primarily by preventing homocysteine accumulation and by preserving genomic methylation integrity. Both nutrients are strongly associated with lower plasma homocysteine levels, with this effect being particularly pronounced in individuals with low folate or vitamin B12 status, indicating that the choline/betaine-dependent pathway serves as a crucial compensatory source of methyl groups [66,67]. Experimental data further show that disruption of choline metabolism produces profound epigenetic instability: choline deficiency diminishes DNMT activity, induces global DNA hypomethylation, and generates the characteristic combination of oncogene hypomethylation (e.g., *c-myc*) and tumor-suppressor hypermethylation (e.g., *p53*, *p16*) that promotes neoplastic transformation [68,69,70,71,72,73,74]. The essential nature of this pathway is highlighted by the spontaneous development of preneoplastic hepatic foci in BHMT-knockout models, where impaired remethylation leads to elevated SAH and widespread methylation defects [75]. Notably, the oncogenic consequences of deficiency are most striking in the liver: choline deprivation is the only known nutritional deficiency capable of independently inducing hepatocellular carcinoma in rodents [76]. This effect arises not only from severe methyl depletion but also from altered lipid-mediated signaling, in which impaired lipoprotein secretion promotes the accumulation of diacylglycerol and chronic protein kinase C activation, disrupting cellular signaling, proliferation–apoptosis balance, and ultimately driving hepatocarcinogenesis [77,78]. In a chemically induced rat liver cancer model, betaine supplementation was shown to alleviate liver injury and attenuate the carcinogenic process. The mechanism involves multiple aspects, including dose-dependently suppressing the upregulation of the proto-oncogene *c-myc* and mitigating the downregulation of the tumor suppressor gene *p16*, thereby reversing carcinogen-induced changes in mRNA levels, while also enhancing the antioxidant capacity of hepatocytes [79]. Collectively, these mechanistic insights suggest that while epidemiologic findings remain inconsistent, adequate choline and betaine status is essential for maintaining methylation fidelity, genomic stability, and proper cellular homeostasis, whereas deficiency potentiates oncogenesis through multiple converging molecular pathways.

Methionine, an essential sulfur-containing amino acid, is central to the one-carbon metabolism network, serving as the immediate precursor for SAM. However, the metabolic impact of methionine intake is highly complex and dose-dependent. Excessive methionine intake can potentially disrupt the crucial balance between SAM and the methylation inhibitor SAH, sometimes by inhibiting the remethylation of homocysteine, consequently impairing overall methylation capacity [80,81]. Due to this intricate metabolic regulation, studies investigating the effects of dietary methionine on methylation status have yielded inconsistent results [6]. Similarly, epidemiological findings regarding methionine and cancer risk are contradictory, echoing the ambiguity seen with other methyl donors [82,83,84]. Although methionine has an important role as a methyl donor, the evidence supporting its potential protective role against the early phases of carcinogenesis remains limited [85]. Notably, studies reporting an inverse association between methionine intake and cancer risk have come almost exclusively from epidemiological research [6]. As a result, despite methionine’s biochemical importance, its role in the initiation of cancer remains insufficiently characterized.

### 3.2. Methyl Donors and Cancer Progression: How Over-Supply Can Drive Tumorigenesis

However, once tumor cells or precancerous lesions have already formed, excessive intake of methyl donor nutrients may become detrimental. One-carbon metabolism plays a crucial role in the de novo synthesis of purines and pyrimidines, which are essential for cancer cell growth [86]. To support their rapid growth and proliferation, cancer cells often upregulate the expression of enzymes involved in one-carbon metabolism, establishing a cancer-specific metabolic signature [26,87,88,89].

As the most extensively investigated methyl donor nutrient, folate has been consistently implicated in promoting cancer progression once malignant or premalignant cells are present. Although studies suggest that folate can protect against the initiation of early neoplastic changes, accumulating evidence indicates that high-dose folic acid may produce opposite effects under certain biological contexts—particularly in individuals with existing precancerous conditions such as inflammatory bowel disease or in relation to cancer recurrence risk [90,91]. This biphasic effect has been clearly demonstrated in animal studies. For example, in two colorectal cancer mouse models [92,93], folate supplementation suppressed tumor initiation and progression in animals that lacked preneoplastic lesions. In contrast, when folate was provided to mice already harboring preneoplastic foci, the same intervention enhanced colorectal tumor development and growth. In rapidly dividing neoplastic cells, where DNA replication is markedly accelerated, disruption of folate-dependent pathways has been shown to impair nucleotide synthesis, ultimately inhibiting tumor expansion. This principle forms the foundation of several antifolate chemotherapies, such as methotrexate and 5-fluorouracil [94]. A More recent study has also introduced novel strategies to inhibit cytoplasmic one-carbon metabolism, thereby reducing nucleotide production and exerting potent antiproliferative effects, including the suppression of cancer cell metastasis [86]. It has also been proposed that high folate consumption may facilitate cancer progression by globally boosting mutation rates, with no apparent bias toward specific mutational signatures [95]. Experimental evidence further supports this concept: transplanted tumors grow more slowly in folate-deficient rats, folate restriction diminishes the growth of virally induced cancers, and lowering dietary folate significantly delays the formation of nerve sheath tumors in transgenic mouse models [96,97,98]. Conversely, provision of folate to established tumors has been associated with an “acceleration phenomenon.” For instance, children with acute leukemia who received folate supplementation exhibited a faster progression of the disease [99]. Collectively, these findings indicate that folate deficiency initiated after neoplastic lesions are established can impede tumor growth or even promote tumor regression, highlighting the stage-dependent nature of folate’s effects on cancer biology.

For vitamin B12, although the concept of a “dual role” similar to that of folate has recently gained attention [7], current evidence is still primarily derived from population-based studies, with a notable lack of mechanistic validation in animal models. Moreover, even the available epidemiological studies face important limitations: vitamin B12 supplementation is often confounded by folate supplementation, making it difficult to determine whether observed associations are truly attributable to vitamin B12 or are instead driven by folate. Another challenge is that these studies rarely distinguish whether participants already harbor precancerous lesions. What can be confirmed, however, is that most cohorts consist of older adults—typically over 60 years of age—an age group more likely to possess undiagnosed precancerous changes or early neoplastic cells. This remains a plausible assumption rather than a verified biological fact, as such conditions are not routinely screened in these studies. For example, a series of RCTs have shown that high-dose folate and vitamin B12 supplementation is associated with a significant increase in overall cancer incidence among older adults, with particularly strong effects observed for gastrointestinal cancers [100,101]. Follow-up methylation profiling further revealed that these elderly populations exhibited DNA methylation abnormalities linked to cancer invasion and metastasis [102]. Specifically, supplementation led to hypermethylation in key tumor suppressor and developmental genes such as *DIRAS3* (a Ras-related tumor suppressor), *NODAL* (a morphogen involved in embryonic patterning and cancer metastasis), and several *HOX* genes (e.g., *HOXB7* and *HOXA4*), which are implicated in cellular invasion and metastatic behavior. These epigenetic alterations, though modest in magnitude, may reactivate potential signaling pathways and promote cancer progression in the presence of pre-existing neoplastic lesions [102]. Given that individuals in this age group are more likely to harbor such epigenetic alterations or undiagnosed precancerous lesions, it is plausible that supplementation with these B vitamins may inadvertently accelerate the progression of pre-existing abnormalities rather than confer preventive benefits.

The concept of a stage-dependent, dual role also extends to vitamin B6, with its active form, PLP, potentially driving progression by supporting the increased metabolic demands of established tumors [57]. This pro-tumorigenic effect is rooted in two distinct mechanisms: metabolic addiction and competition within the tumor microenvironment (TME) [103,104,105]. At the molecular core, PLP acts as an essential cofactor in numerous biosynthetic pathways that fuel rapid cell growth. In cancers such as acute myeloid leukemia, cells exhibit a critical addiction to the vitamin B6 metabolic pathway, selectively upregulating Pyridoxal Kinase (PDXK) to produce high levels of PLP. This high PLP level is exploited to sustain key proliferation pathways, notably by supporting Ornithine Decarboxylase 1 (ODC1) for polyamine synthesis (essential for DNA replication) and Glutamic-Oxaloacetic Transaminase 2 (GOT2) to fuel glutamine-driven biomass production (anaplerosis). Pharmacological or genetic blockade of this PDXK-PLP axis effectively inhibits leukemic cell proliferation in vitro and delays progression in vivo [103,104]. Conversely, an indirect mechanism involves TME competition, as seen in pancreatic ductal adenocarcinoma. Highly metabolic tumor cells actively and aggressively consume available vitamin B6, leading to PLP deprivation in the surrounding TME. This scarcity critically impacts immune surveillance, as anti-tumor Natural Killer (NK) cells require PLP to facilitate the intracellular glycogen breakdown necessary for their activation and cytotoxic functions, effectively dampening the host’s anti-cancer immune response and facilitating tumor progression. This creates a paradoxical situation where vitamin B6 supplementation can inadvertently accelerate tumor growth by feeding the metabolic addiction of cancer cells, while the aggressive PLP consumption by the tumor starves the host’s NK cells of the co-factor they need to mount an anti-cancer defense [105].

Large-scale epidemiologic evidence also raises concerns regarding the potential adverse effects of long-term, high-dose supplementation with certain methyl donor–related B vitamins. The Vitamins and Lifestyle (VITAL) prospective cohort, which followed over 77,000 adults aged 50–76 years for cancer outcomes, reported that prolonged use of supplemental vitamin B6 and vitamin B12 was associated with a substantially elevated risk of lung cancer in men [106]. The strongest associations were observed among individuals with the highest intake categories: men consuming more than 20 mg/day of vitamin B6 or more than 55 μg/day of vitamin B12 for ten years exhibited an approximately 80–100% higher risk of developing lung cancer compared with non-users. The increase in risk was even more pronounced among current male smokers, in whom the hazard ratios approached a threefold elevation for both vitamins. In contrast, no significant associations were observed for women, former smokers, or recent quitters, suggesting sex-specific and exposure-dependent interactions. Mechanistically, these findings are consistent with emerging evidence that excessive provision of one-carbon cofactors may disrupt DNA methylation homeostasis in susceptible individuals. The VITAL cohort results raise the possibility that chronic exposure to supraphysiologic vitamin B6 or vitamin B12 may amplify aberrant methylation changes in precancerous epithelial cells—particularly in the context of continuous carcinogenic stimuli such as tobacco smoke. Androgen-regulated differences in one-carbon metabolism may further contribute to the male-specific effect observed [107]. Collectively, these results suggest that while maintaining adequate vitamin B6 and vitamin B12 status is essential for preserving methylation fidelity and genomic stability, excessive and prolonged supplementation—especially in high-risk populations such as male smokers and older adults—may inadvertently potentiate carcinogenic processes rather than prevent them.

Additionally, evidence demonstrates that dietary methyl-donor deficiency can significantly suppress tumor development during cancer progression, particularly in genetically predisposed models of colorectal neoplasia [108,109,110]. In *Apc*-mutant mice, simultaneous restriction of folate, methionine, choline, and vitamin B12 produces a dramatic and durable reduction in intestinal tumor burden—exceeding 95% in early studies and consistently reducing tumor multiplicity by 50–80% across both the small intestine and colon [108,109]. At the stage of cancer progression, methyl-donor deprivation exerts a multifaceted inhibitory effect on tumor biology. First, it remodels intestinal epithelial homeostasis [108]. Methyl-donor–deficient (MDD) diets shorten crypt length, suppress crypt fission, and markedly decrease proliferative indices (Ki-67 and phospho-Histone H3), while simultaneously enhancing apoptosis in both normal crypts and neoplastic lesions [108,110]. A striking depletion of Dclk1^+^ long-lived crypt cells—considered a population with cancer stem cell potential—suggests that MDD may diminish the pool of tumor-initiating cells, thereby limiting the capacity for neoplastic expansion and progression [109,110]. At the metabolic level, multi-omics profiling reveals that MDD suppresses methionine-cycle flux, sharply lowering methionine and betaine while driving a >100-fold accumulation of homocysteine and a substantial rise in SAH. This shift favors transsulfuration, elevating cystathionine, cysteine, and hypotaurine, collectively reshaping redox balance and correlating tightly with reduced proliferation and increased apoptosis [110]. Beyond one-carbon metabolism, MDD induces unexpected but functionally relevant alterations in additional metabolic pathways. Secondary bile acids including deoxycholic acid and muricholates are significantly reduced, a change consistent with diminished bile-acid–driven epithelial proliferation and inflammation [110]. Furthermore, methyl-donor restriction disrupts carnitine-dependent fatty acid β-oxidation, evidenced by significant depletion of carnitine and a wide panel of acylcarnitines, reduced acetyl-CoA availability, downregulation of CPT1a, CPT2, and PPARα, and mitochondrial abnormalities such as swelling and impaired energy metabolism [110]. This energetic stress likely contributes to reduced proliferative capacity and enhanced epithelial turnover. Importantly, although some metabolic disturbances and adverse effects (e.g., reduced body-weight gain, hepatic steatosis) occur during active depletion, they are largely reversible after methyl-donor repletion—yet tumor protection and certain epithelial alterations persist, indicating durable reprogramming of the intestinal microenvironment [108].

Building upon the efficacy of broad methyl-donor deprivation, specific restriction of methionine unveils a distinct and pervasive metabolic vulnerability in neoplastic cells known as the “Hoffman effect,” where tumors exhibit a heightened dependency on exogenous methionine that often exceeds even the “Warburg effect” of glucose overuse [111]. This “methionine addiction” is driven by excessive transmethylation demands required to sustain oncogenic epigenetic programs, polyamine biosynthesis, and redox homeostasis via GSH production [111,112,113,114,115,116]. This hyperactive transmethylation flux paradoxically diverts methyl groups away from the genome, perpetuating a state of global DNA hypomethylation that fosters genomic instability and aneuploidy, thereby driving tumor heterogeneity and evolution [117,118]. Therapeutic methionine restriction (MR) exploits this addiction by depleting the intracellular SAM pool, which not only erases critical oncogenic histone marks (e.g., H3K4me3, H3K9me3) but can also activate immune surveillance pathways such as the cGAS-STING axis and enhance CD8+ T cell cytotoxicity within the tumor microenvironment [113,119,120,121,122]. Furthermore, MR exploits specific genetic lesions; for instance, cancers harboring methylthioadenosine phosphorylase deletions accumulate methylthioadenosine, rendering them synthetically lethal to methionine adenosyltransferase 2A (MAT2A) inhibition and protein arginine methyltransferase 5 (PRMT5) suppression due to a complete collapse of the salvage pathway [118]. Beyond epigenetic remodeling, MR induces metabolic catastrophe by arresting the cell cycle in the S/G2 phase and triggering ferroptosis through the downregulation of *SLC43A2* and *GPX4*, while simultaneously sensitizing chemoresistant tumors to 5-fluorouracil and radiation by depleting thymidylate synthase and disrupting nucleotide metabolism [113,118]. Consequently, targeted methionine restriction acts not merely as a nutritional intervention but as a potential precision metabolic therapy that dismantles the bioenergetic and epigenetic scaffolding required for cancer progression. To summarize the stage-dependent roles and mechanisms of various methyl-donor nutrients in cancer development, Table 1 provides an integrated overview of their effects on potential molecular targets and functional consequences.

## 4. Conclusions and Future Directions

Methyl-donor nutrients occupy a uniquely paradoxical position in cancer biology. As central regulators of one-carbon metabolism, these nutrients safeguard genomic stability, preserve epigenetic fidelity, and maintain redox and metabolic homeostasis—functions that collectively protect against the initiation of malignant transformation. Evidence from epidemiologic studies, mechanistic investigations, and precancerous-lesion research suggests that adequate levels of folate, vitamin B12, vitamin B6, choline, betaine, and methionine are essential for preventing early epigenomic disruption, limiting DNA damage, and suppressing the earliest steps of carcinogenesis. However, once preneoplastic or malignant cells are established, the same nutrients may instead promote tumor progression by supplying one-carbon units for nucleotide biosynthesis, fueling methylation-dependent silencing of tumor-suppressor pathways, and supporting the heightened metabolic demands and proliferative programs of cancer cells. This dualistic, stage-dependent behavior underscores the complexity of nutritional–epigenetic interactions and highlights the urgent need to move beyond conventional epidemiologic approaches toward mechanistically informed, temporally nuanced frameworks.

In our opinion, future research may prioritize the integration of multi-omics profiling, lineage-tracing models, and temporal intervention studies to disentangle how methyl-donor availability shapes distinct phases of tumor evolution. Mechanistic work is also needed, as the effects of these nutrients remain insufficiently mapped in experimental systems. Furthermore, the potent tumor-suppressive effects observed under methyl-donor restriction and methionine-targeted metabolic interventions warrant further exploration to determine their therapeutic potential, optimal timing, and safety in clinical settings. Additionally, inter-individual variability driven by genetic polymorphisms, age-related epigenetic drift, microbiome composition, and environmental exposures should be incorporated to refine personalized nutritional strategies. Ultimately, translating these insights into clinical practice will require a shift toward precision nutrition, where timing, baseline metabolic status, genetic background, and disease stage collectively guide whether methyl-donor modulation functions as a preventive measure. Such an approach will be promising for leveraging the double-edged nature of methyl donors to improve cancer prevention, risk stratification, and treatment outcomes.

## Figures and Tables

**Table 1:** Stage-dependent roles of individual methyl-donor nutrients in cancer development: key mechanisms, potential molecular targets, and functional consequences.

Methyl-Donor Nutrient	Primary Role in One-Carbon Metabolism	Key Molecular Mechanisms	Representative Molecular Targets/Pathways	Functional Consequences in Cancer Prevention	Functional Consequences in Cancer Progression
Folate	Supplies one-carbon units for nucleotide synthesis and remethylation of homocysteine to methionine	Maintains SAM availability; regulates DNA synthesis and DNA/histone methylation	DNMTs; *LINE-1*; *PDGF-B*; *survivin*; *p16*; *p53*	Preserves genomic stability; prevents global DNA hypomethylation; suppresses oncogene activation and chromosomal instability	Fuels nucleotide biosynthesis in rapidly dividing cells; may enhance tumor growth and recurrence when preneoplastic or malignant cells are present
Vitamin B12	Cofactor for MS linking folate and methionine cycles	Prevents methyl-folate trap; sustains SAM synthesis; stabilizes methylation capacity	MS; SAM/SAH ratio; *p16*; *p53*	Supports effective remethylation; prevents homocysteine accumulation and epigenetic instability in early carcinogenesis	Excessive supplementation may exacerbate aberrant DNA methylation patterns and promote progression in populations with latent neoplasia
Vitamin B6	Cofactor for SHMT and transsulfuration pathway	Maintains nucleotide integrity; prevents uracil misincorporation; supports redox balance	SHMT; CBS; CSE; ODC1; GOT2; NK cell metabolic pathways	Reduces DNA strand breaks; lowers homocysteine; mitigates oxidative stress and inflammation	Supports metabolic addiction of tumor cells (polyamine synthesis, anaplerosis); depletion in TME impairs anti-tumor immunity
Choline/Betaine	Alternative methyl donor via BHMT pathway	Maintains methionine and SAM pools; regulates DNMT activity	BHMT; DNMTs; *c-myc*; *p16*; *p53*	Prevents global DNA hypomethylation; maintains epigenetic fidelity and lipid homeostasis	Excess availability may support methylation-dependent silencing and lipid-mediated oncogenic signaling
Methionine	Direct precursor of SAM	Controls transmethylation flux; regulates epigenetic and redox pathways	MAT2A; PRMT5; histone methylation marks (H3K4me3, H3K9me3); cGAS–STING	Essential for normal methylation and genomic maintenance at physiological levels	Tumor cells exhibit “methionine addiction”; restriction disrupts epigenetic programs, induces metabolic stress and ferroptosis

## Data Availability

No new data were created or analyzed in this study. Data sharing is not applicable to this article.
